# Converging Work-Talk Patterns in Online Task-Oriented Communities

**DOI:** 10.1371/journal.pone.0154324

**Published:** 2016-05-03

**Authors:** Qi Xuan, Premkumar Devanbu, Vladimir Filkov

**Affiliations:** 1 Department of Automation, Zhejiang University of Technology, Hangzhou, China; 2 Department of Computer Science, University of California Davis, Davis, CA, United States of America; Tianjin University of Technology, CHINA

## Abstract

Much of what we do is accomplished by working collaboratively with others, and a large portion of our lives are spent working and talking; the patterns embodied in the alternation of working and talking can provide much useful insight into task-oriented social behaviors. The available electronic traces of the different kinds of human activities in online communities are an empirical goldmine that can enable the holistic study and understanding of these social systems. Open Source Software (OSS) projects are prototypical examples of collaborative, task-oriented communities, depending on volunteers for high-quality work. Here, we use sequence analysis methods to identify the work-talk patterns of software developers in online communities of Open Source Software projects. We find that software developers prefer to persist in same kinds of activities, i.e., a string of work activities followed by a string of talk activities and so forth, rather than switch them frequently; this tendency strengthens with time, suggesting that developers become more efficient, and can work longer with fewer interruptions. This process is accompanied by the formation of community culture: developers’ patterns in the same communities get closer with time while different communities get relatively more different. The emergence of community culture is apparently driven by both “talk” and “work”. Finally, we also find that workers with good balance between “work” and “talk” tend to produce just as much work as those that focus strongly on “work”; however, the former appear to be more likely to continue to be active contributors in the communities.

## Introduction

A great deal of adult life is spent working. We work to create materials that fulfill human needs, to develop advanced technologies, to govern, heal, and teach each other, etc. Our work is often collaborative, and often involves repeated activities: i.e., we commute, work, collaborate with others, etc. Collaborations involve both *talking* and *working*. We get some work done, talk with our colleagues to socialize, learn, or further co-ordinate tasks, and then work some more. The recurrent practices constitute patterns of activities that can be used to characterize individuals, cluster them, and then predict their future behaviors; this has potential applications in various areas including crime control [1, 2], traffic forecasting [3, 4], and marketing [5, 6]. In this paper, we will focus on the two most basic activities, i.e., work and talk. Talking, or communication, plays a key role in the coordination between co-operating individuals. As a result, communication traces are commonly used to infer the social networks as the discrete spaces to study the dynamics of many other activities [7–9].

Sequence analysis, which has long history of being useful in molecular biology [10], has been, as of recently, also used in social science [11, 12], where researchers investigate life courses [13], and career trajectories [14]. Whereas DNA sequences are curled up in three-dimensional space, social events are arranged according to their time of occurrence. Due to our interest in social phenomena mostly local in time, the positions of social events in a sequence refer to relative, rather than absolute, time points. In bioinformatics, a number of global and local sequence alignment methods are used to compare the molecules’ genetic similarity within and across different organisms, so as to elucidate their biological functions [15, 16]. Here we adopt a local alignment method to find and enumerate short patterns in work-talk (W-T) sequences of different people in online communities. We use these short W-T pattern counts as data points for modeling human behavior using hidden Markov models (HMMs) [17]. The goodness of fit of these models are established via their ability to predict the numbers of larger patterns in the sequences [15].

In collaborative communities there is interplay between work and talk activities, resulting in meaningful W-T sequence patterns that can be used to characterize different individuals. E.g., the simplest distinguishing W-T pattern for an individual is that they either tend to work continuously on the shared product, i.e. the sequence WWWW…, or talk continuously to co-ordinate work with others, and strengthen relationships, i.e. the sequence TTTTT…. More complex patterns are a combination of the two. If the W-T patterns are shared between people, then whole communities can also be characterized along those patterns as having a shared “community culture”, in this case a work culture. This connotation of “culture” is consistent with Etzioni’s notion [18]: “the set of assumptions shared by members of a societal unit which sets a context for its view of the world and itself”. It is known that community culture plays an important role in innovation [19], the quality of work-products [20], and can facilitate the decision-making [21]. Recent studies on collaboration reveal that community size, team assembling mechanisms, and team structure have significant effects on team performance [22–24]. Thus, quantitatively characterizing the community culture is of much interest and provides novel insights for collaboration especially when it can be related to individual productivity and work efficiency.

To quantitatively study the emergence of work culture in online task-oriented groups we built an analytic framework for analysis of patterns jointly emerging along two basic collaborative dimensions, work and talk. Particularly, different from the previous studies mostly relied on talk activities to generate social networks [7–9], here we treat both activities equally as events sequenced over time, and use sequence analysis methods and stochastic models to reveal and contrast W-T patterns of individuals in online task-oriented communities. Here, we use OSS communities, where abundant data is publicly available [25–27], to study the W-T patterns of software developers for three main reasons. First, the work in OSS communities is easy to observe, and most of the talk activities are meaningfully related (because of community norms) to work activities; this simplifies the observation of functional W-T patterns. Second, the work and talk activities in OSS communities are always archived [28], so they are readily collected for analysis. Finally, performance properties, such as productivity, in terms of number of lines of code (LoC) written, can also be measured using the state of the produced software.

In this paper, we make the following specific contributions. We first propose two-state dynamic hidden Markov models (HMMs) as abstractions for software developers’ work-talk behavior over time, and apply them on W-T sequences derived from Apache Software Foundation OSS projects. By characterizing each developer using their corresponding HMM parameters, we find evidence for community culture in OSS projects: developers in the same community tend to have more similar W-T patterns than those from different ones, and this pattern-affinity strengthens with time. We also observed that developers who have balanced W-T patterns are just as productive as those who work more continuously (fewer “talk” interruptions), but the former tend to stay active longer; this suggests that W-T balance is important to sustain OSS communities. Moreover, we create social and cooperation networks, and find that the convergence of W-T patterns between a given pair of developers appears to be re-enforced by both talk- and work-related interactions. This indicates that the emergence of a community W-T culture is apparently driven by both “work” and “talk” activities, and may offer a novel perspective on the co-evolving mechanisms of socio-technical, interdependent networks [29–31].

## Materials and Methods

**Fig 1:**
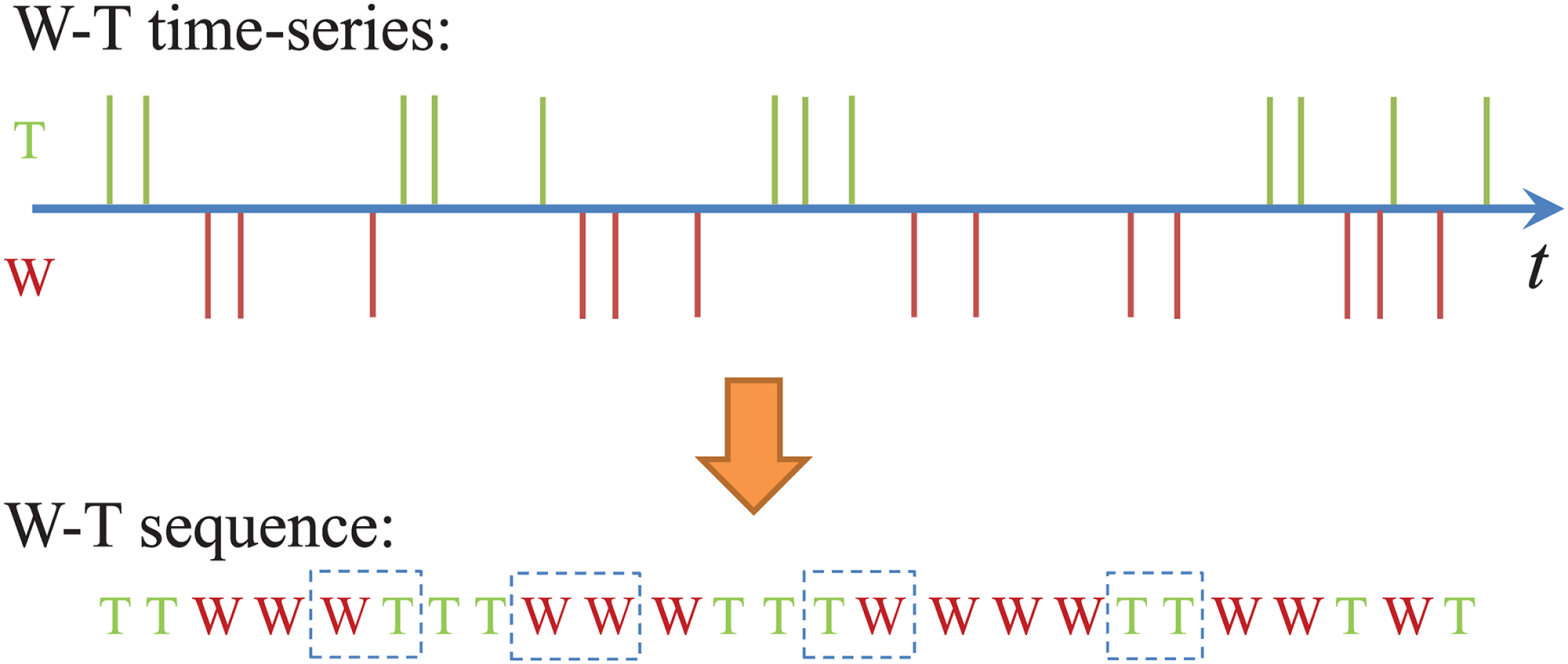
A multiple time-series of work and talk activities and the corresponding W-T sequence. The four different two-patterns, i.e., WW, WT, TW, and TT, are marked by the dashed rectangles.

We follow the definitions of *work* and *talk* in software engineering [28] and collected these activities from 31 OSS communities in the *Apache Software Foundation* on March 24th, 2012. In each community, there are several volunteer developers who contribute by committing to files, i.e., adding or removing software code; these activities are recorded in a Git repository and are our “work events”, or “W”s. OSS developers use *developer mailing lists* to share programming knowledge and coordinate with others in the project. We record sent emails of a developer as “talk events”, or “T”s (the received emails are included in the talk activities of others). Using this data, a W-T sequence of work and talk activities, as shown in Fig 1, can be recorded for each developer. Note that messages may be automatically posted to a mailing list in an OSS community to inform others when some work is completed. We exclude such trivial talk activities and only consider response emails [32, 33] which make up about 73% of all messages. We also use a semi-automatic approach to solve the problem of multiple aliases [32].

We pre-process the W-T sequence data in several ways. To ensure a sufficient number of samples to reliably compare the W-T patterns between pairs of developers in the same or from different communities, we select a subset of “top developers” with sequences including at least 500 work and talk activities, and a subset of communities with at least 5 such developers. We acknowledge a risk of left-censorship of both work & talk activities, if any OSS communities did not archive their emails, or if they had used different version control systems before they moved to Git, some early data could be lost. Besides, it is known that many individuals need to first earn social capital in the OSS community by communicating with others before they are accepted as developers [34, 35]. As a result, we often observe long, pure work (resp. talk) subsequences before the first talk (resp. work) activity of a developer. In this study, we remove these trivial prefixes of pure work or talk activities, i.e., we only consider W-T sequences starting from the first work (resp. talk) activity if it occurred after a talk (resp. work) activity.

The above pre-processing of the data yielded 14 communities with 120 “top developers”. The full data is available at: https://dx.doi.org/10.6084/m9.figshare.3181555. Some basic properties of those OSS projects are shown in Table 1. Besides developers, there we also list the number of active users (including developers) in each community. These users might not directly change files, but they may contribute to the communities by other ways, such as report bugs etc.

**Table 1:** Basic properties of the fourteen OSS communities.

Communities	Description	Time frame	#Users	#Devs	#Top devs	#Files
Activemq	Integration patterns server	2005/12/12–2012/03/16	2012	28	6	16788
Ant	Build tool	2000/01/13–2012/03/16	1402	44	9	11620
Axis2_c	Web services engine	2004/02/03–2012/03/15	582	24	8	10262
Axis2_java	Web services engine	2001/01/30–2012/03/19	3738	72	15	129978
Camel	Integration framework	2007/03/19–2012/03/17	805	31	6	36965
Cxf	Web services framework	2005/07/22–2012/03/16	427	45	7	37867
Derby	Database management system	2004/08/10–2012/03/22	1118	35	16	6563
Lucene	Search software	2001/09/11–2012/03/23	2102	41	14	6674
Mahout	Machine learning library	2008/01/15–2012/03/23	533	15	6	5123
Nutch	Web search software	2005/01/25–2012/03/22	556	16	6	3072
Ode	Web services	2006/02/18–2012/03/22	365	17	6	11006
Openejb	Container system and server	2002/01/18–2012/03/22	169	38	5	43960
Solr	Enterprise search platform	2006/01/20–2011/03/01	825	19	8	8534
Wicket	Web application framework	2004/09/21–2012/03/21	539	24	8	48045

### Finding Surprising Sequence Patterns

A *G*-pattern in a sequence over the alphabet {W, T} is a subsequence of length *G*. There are total 2^*G*^ possible different *G*-patterns. Typically, the length of a pattern is much shorter than the length of the given sequence. In our study we focus on 2-patterns and 3-patterns. Given a sequence *θ* = {*s*_1_, *s*_2_, …, *s*_*h*_} over {W, T}, we count the occurrence of each of the 2^*G*^ patterns, by rolling a window of size *G* over the sequence, and incrementing the count for the pattern we find. For instance, in the W-T sequence shown in Fig 1, the four possible 2-patterns, WW, WT, TW, and TT, occur eight, five, five, and six times, respectively.

To assess the probability that a pattern occurs by chance, we create a null (baseline) model by randomizing the observed W-T sequence so as to preserve the proportion of work to talk activities. This can be achieved, e.g., by using the R’s [36] sample() function on the sequence indexes. Then, the preference for pattern *P* in the observed sequence, *θ*, over the randomized sequence, θ*, is calculated by the relative difference between the counts for that pattern, *C*_*P*_ and $C_{P}^{*}$, in the respective sequences,

$$\begin{array}{c} \lambda_{P}=\frac{C_{P}-\left\langle C_{P}^{*}\right\rangle }{\left\langle C_{P}^{*}\right\rangle }\times 100\%. \end{array}$$ (1)

For $\langle C_{P}^{*}\rangle$, we generated 100 randomized sequences for each observed one. For each pattern *P* in a sequence, we also calculate its *Z*-score [37] as $Z=\lambda_{P}\langle C_{P}^{*}\rangle /\varsigma$, where *ς* is the standard deviation of the pattern counts in θ*. Larger |*Z*| values indicate more surprising observed counts.

### Hidden Markov Model

**Fig 2:**
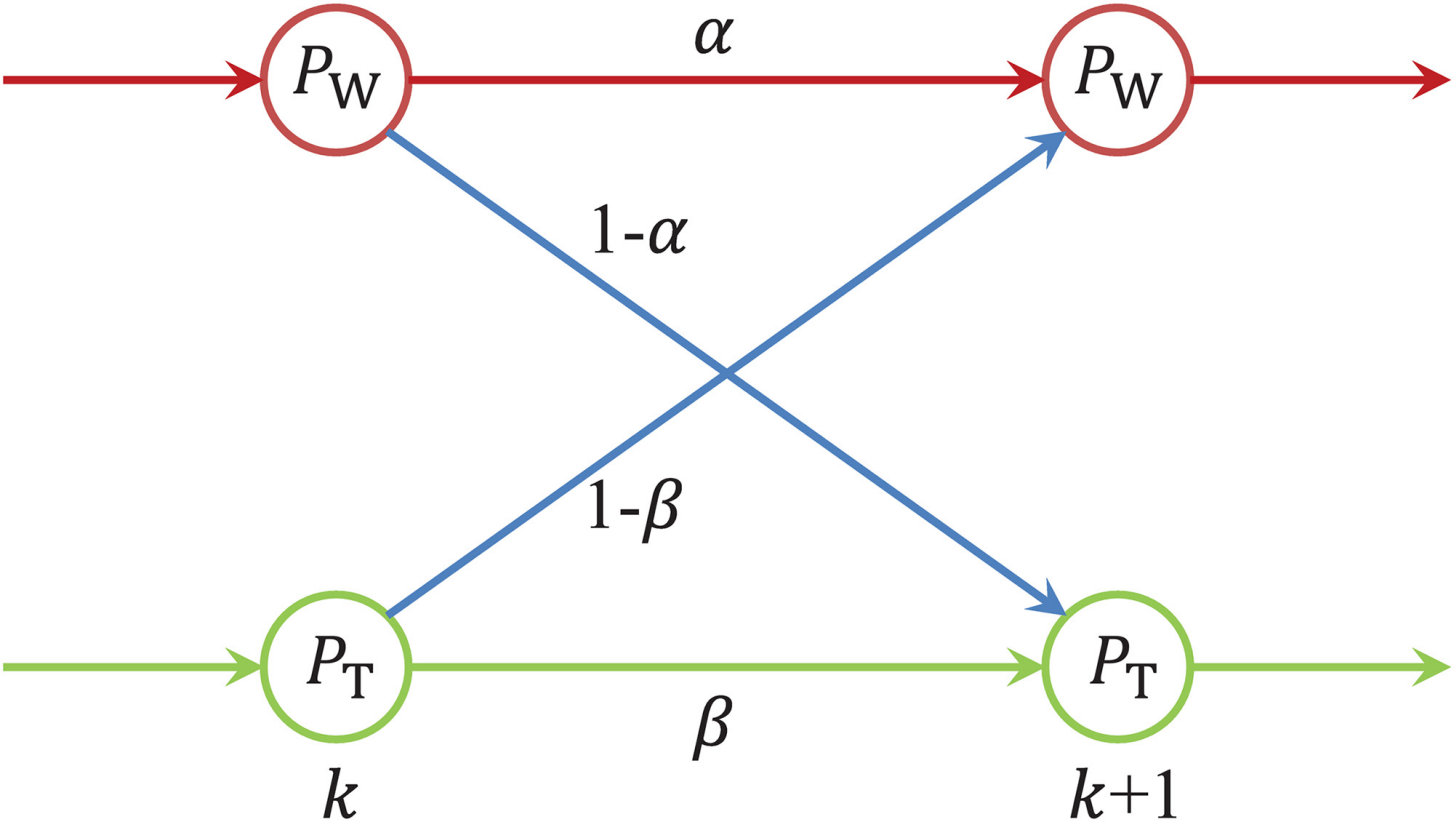
An HMM with two states, i.e., “work” and “talk”, denoted by “W” and “T”, respectively. The model is used to explain the W-T patterns of developers in different communities.

A Hidden Markov Model, HMM, is a simple stochastic model used to abstract behavior involving several different states and transitions among them. To model developers and their work-talk behavior, we use an HMM with two states, “work”, “W”, and “talk”, “T”, and transitions between them corresponding to either continuing to perform the same activity, W followed by a W or T followed by a T, or switching activities, W followed by a T, and vice versa. The parameters *α* and *β*, representing the conditional transition probabilities *P*(W|W) and *P*(T|T), respectively. The HMM diagram is shown in Fig 2.

If we denote by *P*_W_(*k*) and *P*_T_(*k*) the probabilities that work, resp. talk, happen at time step *k*, then for the next time point we have

$$P_{\text{W}}\left(k+1\right)=\alpha P_{\text{W}}\left(k\right)+\left(1-\beta \right)P_{\text{T}}\left(k\right),$$ (2)

$$P_{\text{T}}\left(k+1\right)=\left(1-\alpha \right)P_{\text{W}}\left(k\right)+\beta P_{\text{T}}\left(k\right),$$ (3)

where *α* and *β* are the transition probabilities. We note here that while *α* and *β* could evolve with time, they don’t change much between successive activities, therefore we can consider them as constants in the sequences with certain lengths. Thus, Eqs (2) and (3) can be approximated for continuous time, *τ*, and then transformed to the following more compact matrix form:

$$\begin{array}{c} \dot{P}\left(\tau \right)=\left[\begin{array}{cc} \alpha -1 & 1-\beta \\ 1-\alpha & \beta -1 \end{array}\right]P\left(\tau \right), \end{array}$$ (4)

with *P*(*τ*) = [*P*_W_(*τ*), *P*_T_(*τ*)]^*T*^. By solving Eq (4), we have

$$\begin{array}{c} P\left(\tau \right)=D_{1}\left[\begin{array}{c} 1-\beta \\ 1-\alpha \end{array}\right]+D_{2}\left[\begin{array}{c} 1\\ -1 \end{array}\right]e^{(\alpha +\beta -2)\tau }, \end{array}$$ (5)

where *D*_1_ and *D*_2_ are some constants. The fractions of work and talk activities, *P*_W_ and *P*_T_, in a sequence with length *L* can be estimated by

$$\begin{array}{ccc} \left[\begin{array}{c} P_{W}\\ P_{T} \end{array}\right] & = & \frac{1}{L}\int_{0}^{L}P\left(\tau \right)d\tau . \end{array}$$ (6)

By substituting Eq (5) into Eq (6), we have

$$\begin{array}{ccc} \left[\begin{array}{c} P_{W}\\ P_{T} \end{array}\right] & = & \frac{D_{1}}{(2-\alpha -\beta )L}\left[\begin{array}{c} 1\\ -1 \end{array}\right]\left[1-e^{(\alpha +\beta -2)L}\right]\\ & + & D_{2}\left[\begin{array}{c} 1-\beta \\ 1-\alpha \end{array}\right]. \end{array}$$ (7)

In the right side of Eq (7), the first term is negligible when the sequence is long enough, considering *α* + *β* < 2. Since it is always satisfied *P*_W_ + *P*_T_ = 1, we have

$$\begin{array}{c} P_{W}=\frac{1-\beta }{2-\alpha -\beta },\;P_{T}=\frac{1-\alpha }{2-\alpha -\beta }, \end{array}$$ (8)

which are fully determined by the two parameters in the model. Then, the probabilities for the four different two-patterns in the sequence, in terms of *α* and *β*, are given by:

$$P_{\text{WW}}=\alpha P_{\text{W}}=\frac{\alpha \left(1-\beta \right)}{2-\alpha -\beta },$$ (9)

$$P_{\text{WT}}=\left(1-\alpha \right)P_{\text{W}}=\frac{\left(1-\alpha \right)\left(1-\beta \right)}{2-\alpha -\beta },$$ (10)

$$P_{\text{TW}}=\left(1-\beta \right)P_{\text{T}}=\frac{\left(1-\alpha \right)\left(1-\beta \right)}{2-\alpha -\beta },$$ (11)

$$P_{\text{TT}}=\beta P_{\text{T}}=\frac{\left(1-\alpha \right)\beta }{2-\alpha -\beta },$$ (12)

Intuitively, larger *α* and *β* means higher proportions of WW and TT patterns, respectively, in the sequence. Furthermore, the probabilities for longer patterns can be calculated similarly, once the model parameters *α* and *β* are estimated from Eqs (9) to (12). It is important to note that for the randomized W-T sequences generated by the null model, the current state is independent from the previous state, thus we have *α* = 1 − *β*, i.e., *α* + *β* = 1. In this case, *α* and *β* are equal to the fractions of work and talk activities, respectively.

Based on the above model, we have the following solutions for the parameters:

$$\begin{array}{c} \alpha =\frac{P_{\text{WW}}}{P_{\text{WW}}+P_{\text{WT}}},\;\beta =\frac{P_{\text{TT}}}{P_{\text{TT}}+P_{\text{TW}}}, \end{array}$$ (13)

where *P*_WW_, *P*_WT_, *P*_TW_, and *P*_TT_ denote the probabilities of the four different two-patterns for each developer, and can be estimated from the counts of the four different two-patterns as long as the corresponding W-T sequence is sufficiently long. Thus, this HMM is fully determined by the numbers of the four different two-patterns.

### Hazard Modeling

To study the tenure, or survival time, of developers in the projects (time from joining until leaving) in terms of the HMM parameters *α* and *β*, we use survival analysis, which enables modeling of outcomes in the presence of censored data. In our case the censoring is due to the uncertainty that long time periods without activities may or may not indicate that a developer has left the community. Generally, survival analysis involves calculating the Hazard rate [38], defined as the limit of the number of events per *δt* time divided by the number at risk, as *δt* → 0. Supposing a developer does not leave the community until time Γ, the Hazard rate is given by

$$\begin{array}{c} h\left(t\right)=\lim_{\delta t\to 0}\frac{P(t\leq \Gamma <t+\delta t|t\leq \Gamma )}{\delta t}. \end{array}$$ (14)

Our primary interest is the survival function defined as *S*(*t*) = *P*(*t* < Γ), which can be calculated from Eq (14) by

$$\begin{array}{c} S\left(t\right)=e^{-\int_{0}^{t}h\left(\tau \right)d\tau }. \end{array}$$ (15)

Suppose *α* or *β* can influence the survival time, then we adopt the Cox model [39] to define the Hazard rate *h*(*t*) by

$$\begin{array}{c} h\left(t\right)=h_{0}\left(t\right)e^{bx}, \end{array}$$ (16)

with *h*_0_(*t*) describing how the hazard changes over time at baseline level of covariate *x*, either *α* or *β*. Here we focus on the hazard ratio *h*(*t*)/*h*_0_(*t*) to see whether increasing the covariate will significantly increase or decrease the survival time, e.g., *b* > 0 means that the individuals of larger *x* will have statistically shorter survival times.

## Results

We begin by studying two-pattern preference in developer’s behavior. Given an observed W-T sequence for each person, we count in it the occurrences of all four two-patterns, and derive the preference for each, denoted by *λ*_*i*_, *i* = 1, 2, 3, 4, respectively, in the real sequences as compared to random ones as described above. We find that, on average, for all developers, *λ*_1_ = 148.9% and *λ*_4_ = 40.5%, while *λ*_2_ = −38.0% and *λ*_3_ = −38.6%, i.e., WW and TT are positively enriched, while WT and TW are negatively enriched. We find that |*Z*| > 5 in 462 out of 480 cases (120 developers times 4 two-patterns), indicating that most of the observed counts are surprising. These suggest that developers much prefer to persist with one activity-type, rather than switch frequently between activities.

**Fig 3:**
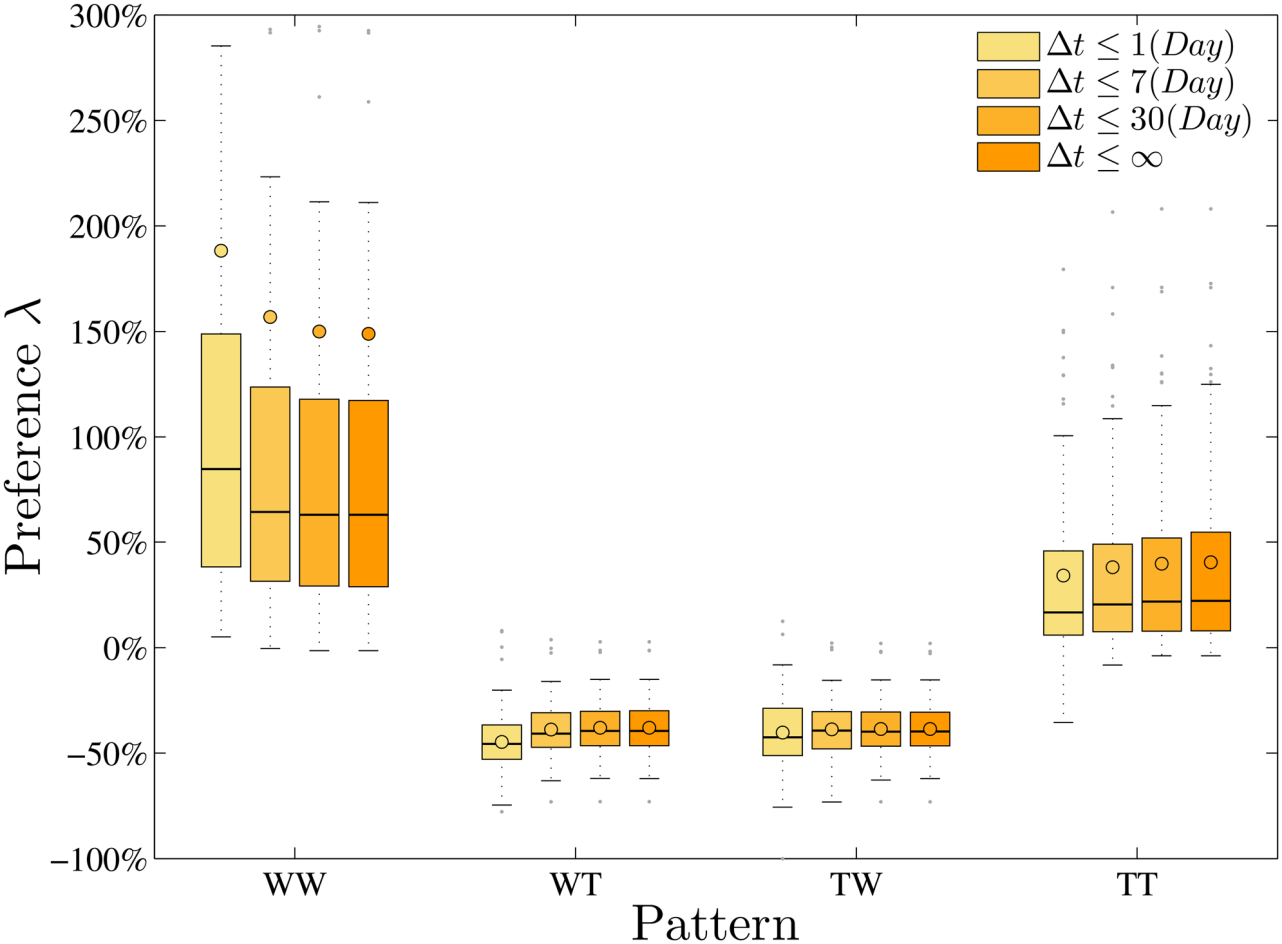
The box-and-whisker diagram for the preferences of the four different two-patterns in the real W-T sequences under the different time-interval conditions by comparing with the random ones.

It may be argued that two successive activities should not be considered as a two-pattern if the time interval between them is relatively long, e.g., longer than one month. To show that our method is robust with respect to time-scale, we also calculate the relative difference by varying the thresholds for the time-intervals over which we consider the two-patterns. We vary the thresholds, denoted by *ξ* = 1, 7, 30 (days), and only the patterns with intervals ≤ *ξ* are considered. The results are shown in Fig 3, where we can see that WW and TT patterns are always much more preferred than WT and TW patterns in the real sequences under thresholds varying from one day to one month. Interestingly, we also find a slight trend that the WW pattern becomes more preferred, and the TT pattern less preferred, when we exclude more repeated activities with relatively shorter time intervals (and thus a smaller *ξ*). Since the number of these long time-interval patterns is relatively small (2.2% and 0.3% for *ξ* = 7 and *ξ* = 30, respectively), this slight trend still indicates that developers are more likely to start and end a repeated and relatively compressed work sequence with talk activities, viz., talk activities plays important role in enabling new tasks (work activities) in these online communities.

### Emergence of Community Culture

**Fig 4:**
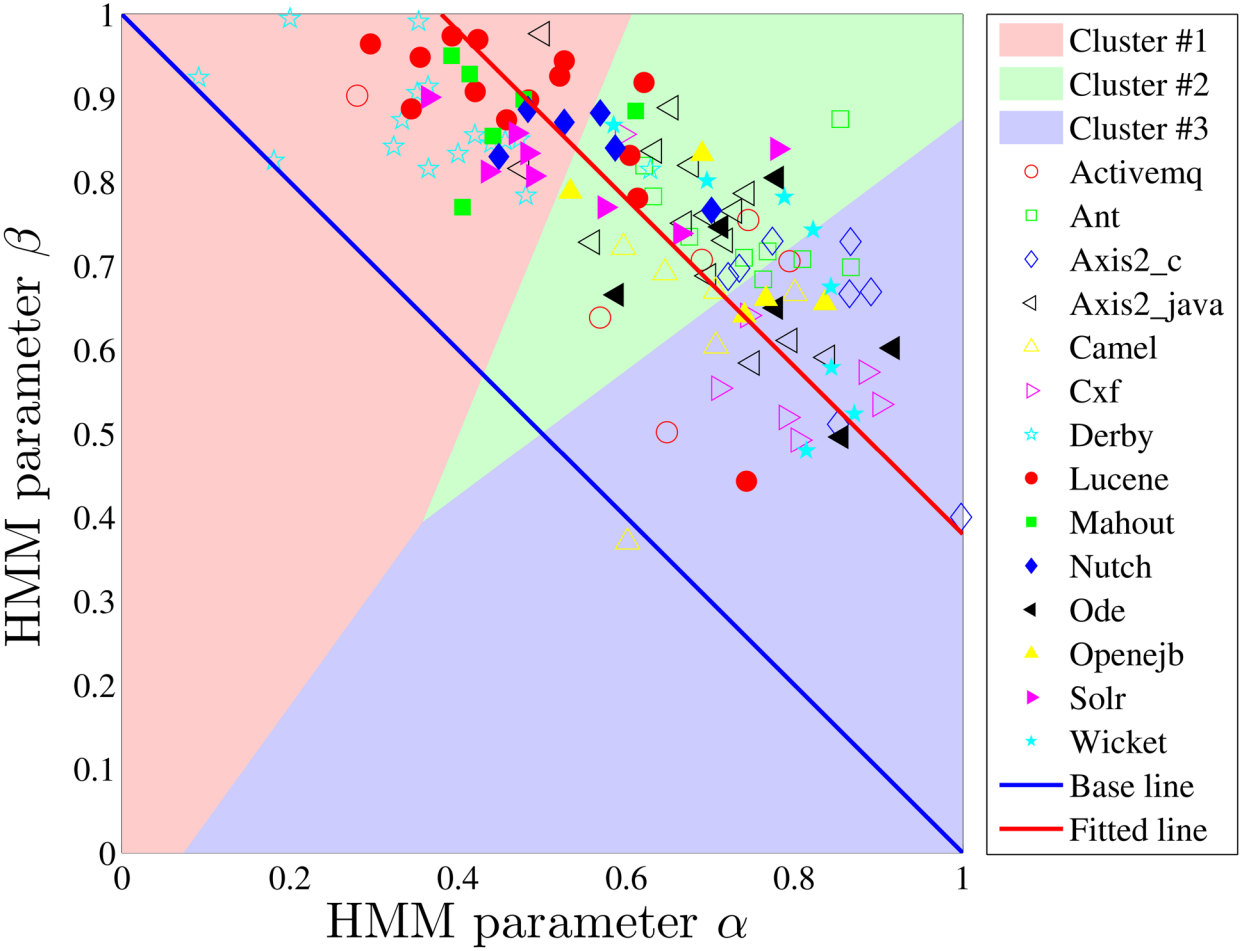
Visualization of developers on *α*-*β* plane by considering their whole sequences, where developers are points and those of the same communities are marked by the same symbols. The parameters are grouped into three clusters by the “K-means” method. The base line is formed by the HMM parameters of the random W-T sequences with different fractions of work activities. The points are fitted by the linear function *α* + *β* = *ε*, with *ε* = 1.38.

We use HMMs, described above, as two parameter, *α* and *β*, models of software developers’ work-talk behavioral patterns. To validate the use of HMMs, we check their efficacy in predicting the counts of longer patterns, e.g., three-patterns. We find that the HMMs do predict the numbers of all the eight three-patterns with significantly smaller relative errors (*p* = 1.8 × 10^−16^ on average) than the random mechanism for the developers we studied, i.e., 14.5% versus 67.4% on average. We characterize each developer with the parameters *α* and *β* coming out of the HMM fitted to their W-T sequence. Those *α* and *β* can, then, be compared across developers and communities. To study the work-talk behavior of developers within and between communities, we first visualize all (*α*, *β*) pairs in the *α* − *β* plane, as shown in Fig 4, where the developers of the same communities are marked by the same symbols. Evidence of clustering is visually apparent: the points representing the developers in the same communities are indeed closer to each other when compared with those from different communities. We further divided all the developers into three groups by the k-means method [40], and find that most developers in the same communities are centralized in one of three clusters, rather than uniformly distributed in all the three, which indicates different community cultures that emphasize continuous work (cluster #3), talk (cluster #1), or both (cluster #2), respectively. Here, we also provide the baseline formed by the HMM parameters of the W-T sequences that are generated by the random mechanism with different fractions of work activities. Since this baseline must satisfy *α* + *β* = 1, and almost all the points being above this based line validates again the preferred patterns WW and TT in all communities.

**Fig 5:**
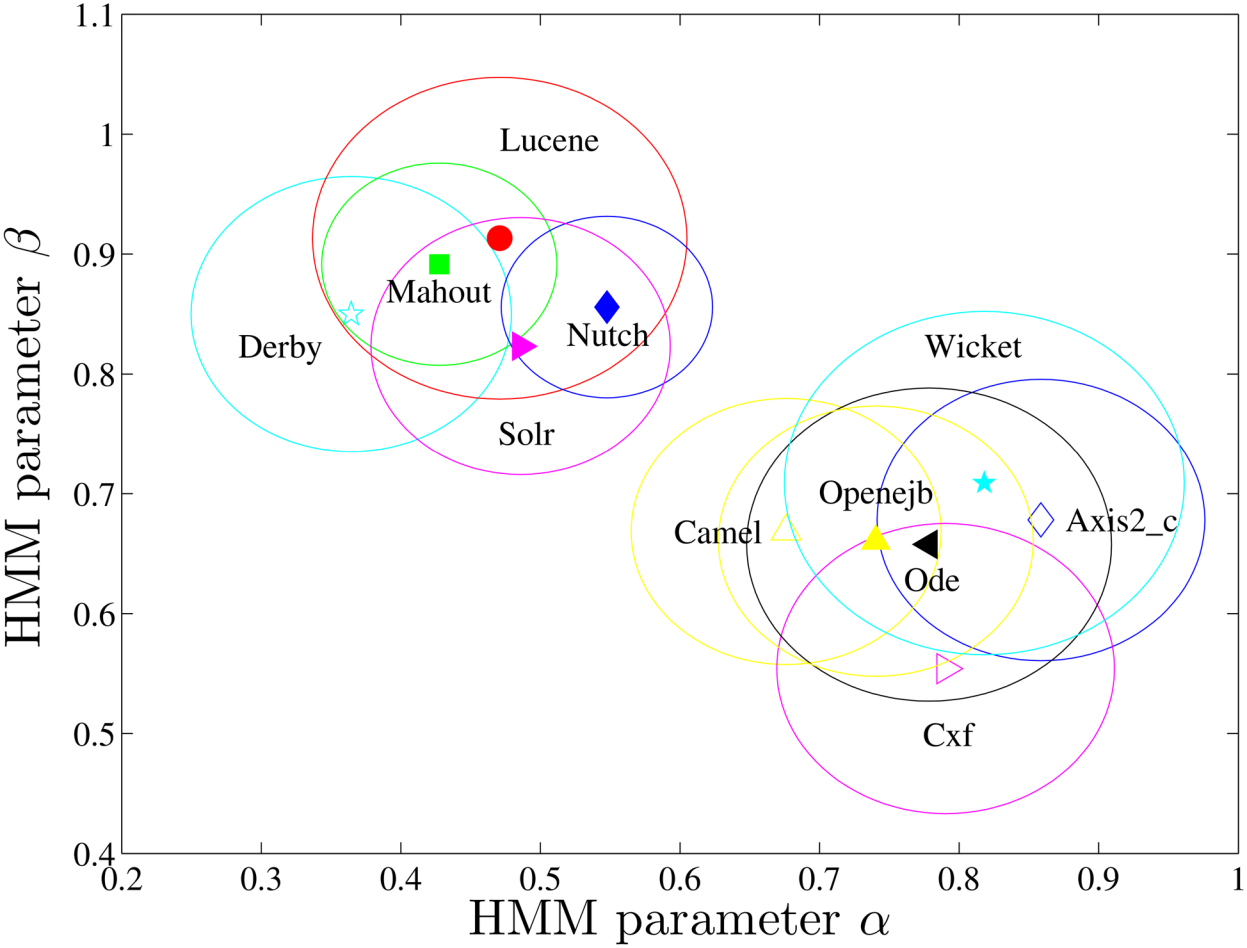
The centers and the respective diversities (the large circles) of the eleven communities on *α* − *β* plane, defined as the medians of the HMM parameters of their developers and the average distances of HMM parameters between the developers and the corresponding centers, respectively.

More specifically, most developers (≥ 50%) in *Derby*, *Lucene*, *Mahout*, *Nutch*, and *Solr* belong to cluster #1, which corresponds to mostly talk activities (high *β*), while most of the developers in *Axis2_c*, *Camel*, *Cxf*, *Ode*, *Openejb*, and *Wicket* belong to cluster #3, corresponding to mostly work activities (high *α*). As a whole, we define the center of a community in *α* − *β* plane by the median of the HMM parameters of the developers in it, then calculate its diversity by the average distances of HMM parameters between the developers and the center, as shown in Fig 5 for the above 11 communities. It is interesting to find that the communities sharing similar W-T patterns also belong to similar domains (description in Table 1). For example, *Lucene*, *Nutch*, and *Solr* are all about “search” and they are intrinsically related to each other, just like the introduction of *Nutch* on its web site: “Stemming from Apache *Lucene*, Apache *Nutch* now builds on Apache *Solr* adding web-specifics”. Besides, *Axis2_c*, *Cxf*, and *Ode* are all about “services”, while each of *Camel*, *Cxf*, and *Wicket* is a software framework that provides a shared architecture for class of applications.

More formally, if we denote by *α*_*i*_ and *β*_*i*_ the HMM parameters of developer *d*_*i*_, we can calculate the Euclidean distance of HMM parameters between two developers *d*_*i*_ and *d*_*j*_ by

$$\begin{array}{c} \rho_{ij}=\sqrt{{\left(\alpha_{i}-\alpha_{j}\right)}^{2}+{\left(\beta_{i}-\beta_{j}\right)}^{2}}, \end{array}$$ (17)

as a quantitative metric for the similarity between the W-T patterns of developers, i.e., the shorter the distance between them, the more similar the W-T patterns of the two developers. Then, we compare the distances of HMM parameters between all pairs of developers in the same communities with those between pairs of developers from different communities, and find that the former list of distances are significantly shorter (*p* = 0) than the later ones. These qualitative and quantitative analysis lend support to using the HMM parameters as a reasonable proxy for the way the interplay of work and talk testify to community culture.

**Fig 6:**
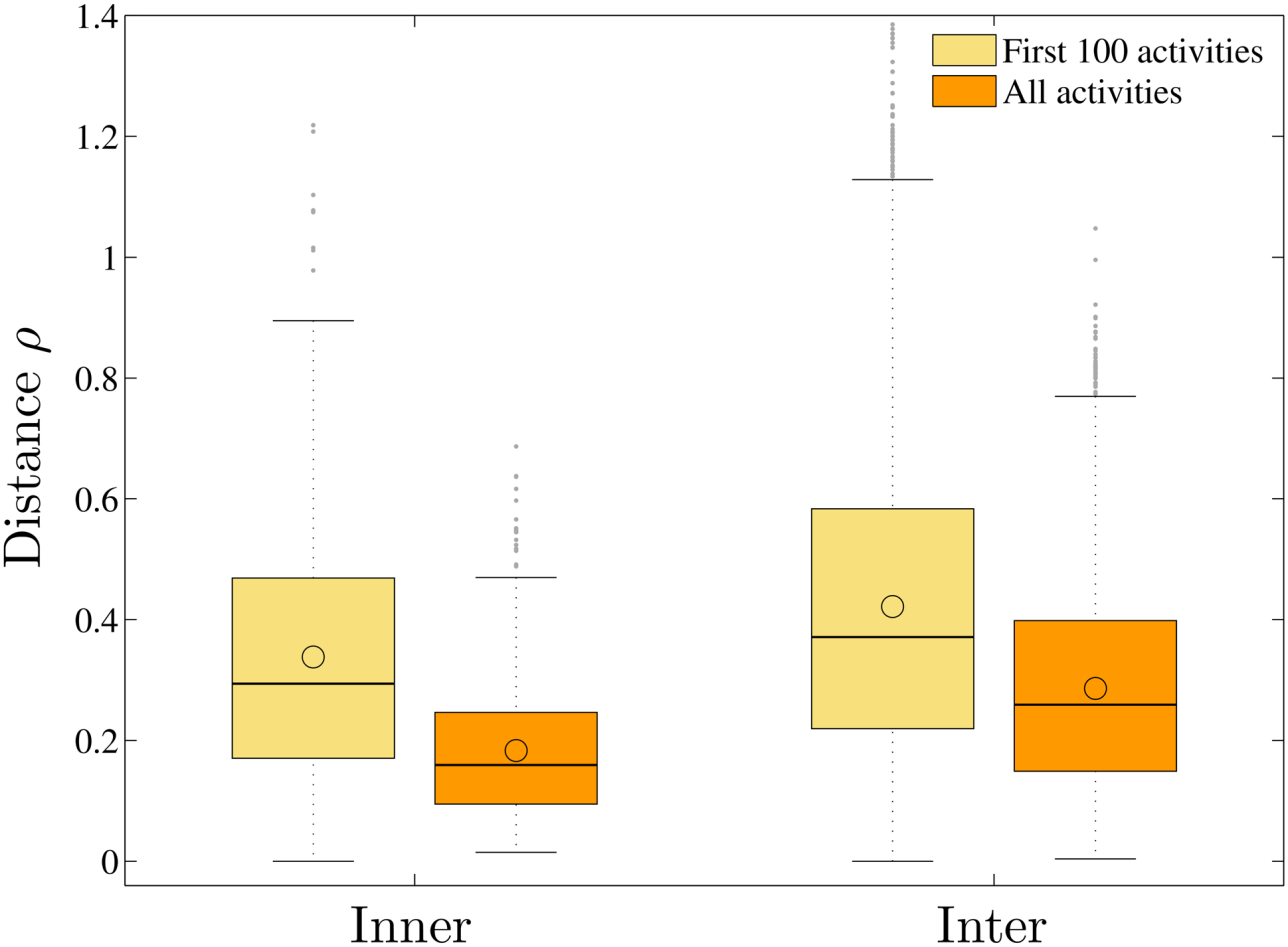
The box-and-whisker diagrams for the distances of the HMM parameters of the first 100 activities and those of the whole W-T sequences between pairs of developers inner and inter communities.

**Fig 7:**
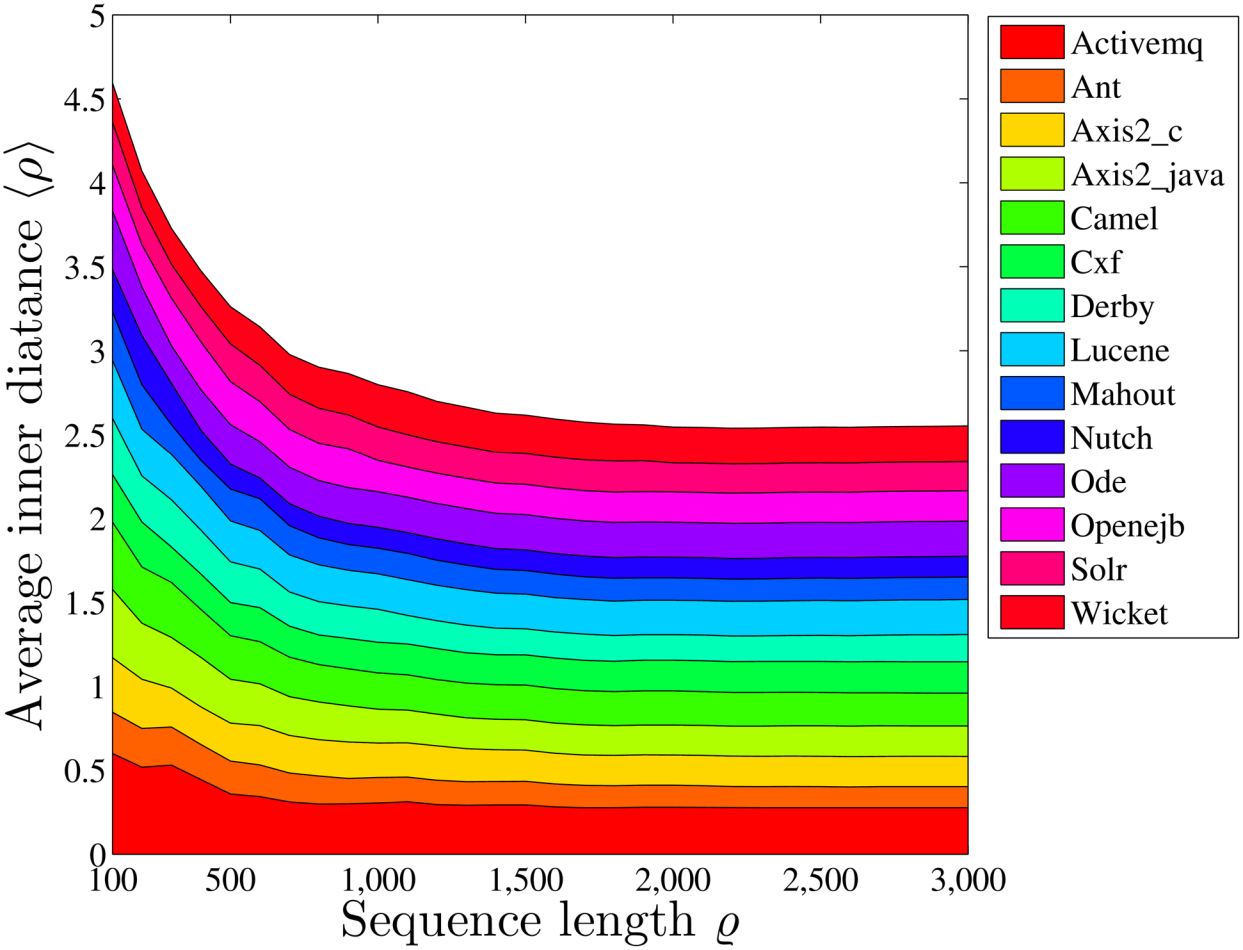
The average inner distances of HMM parameters between pairwise developers for the fourteen communities.

**Fig 8:**
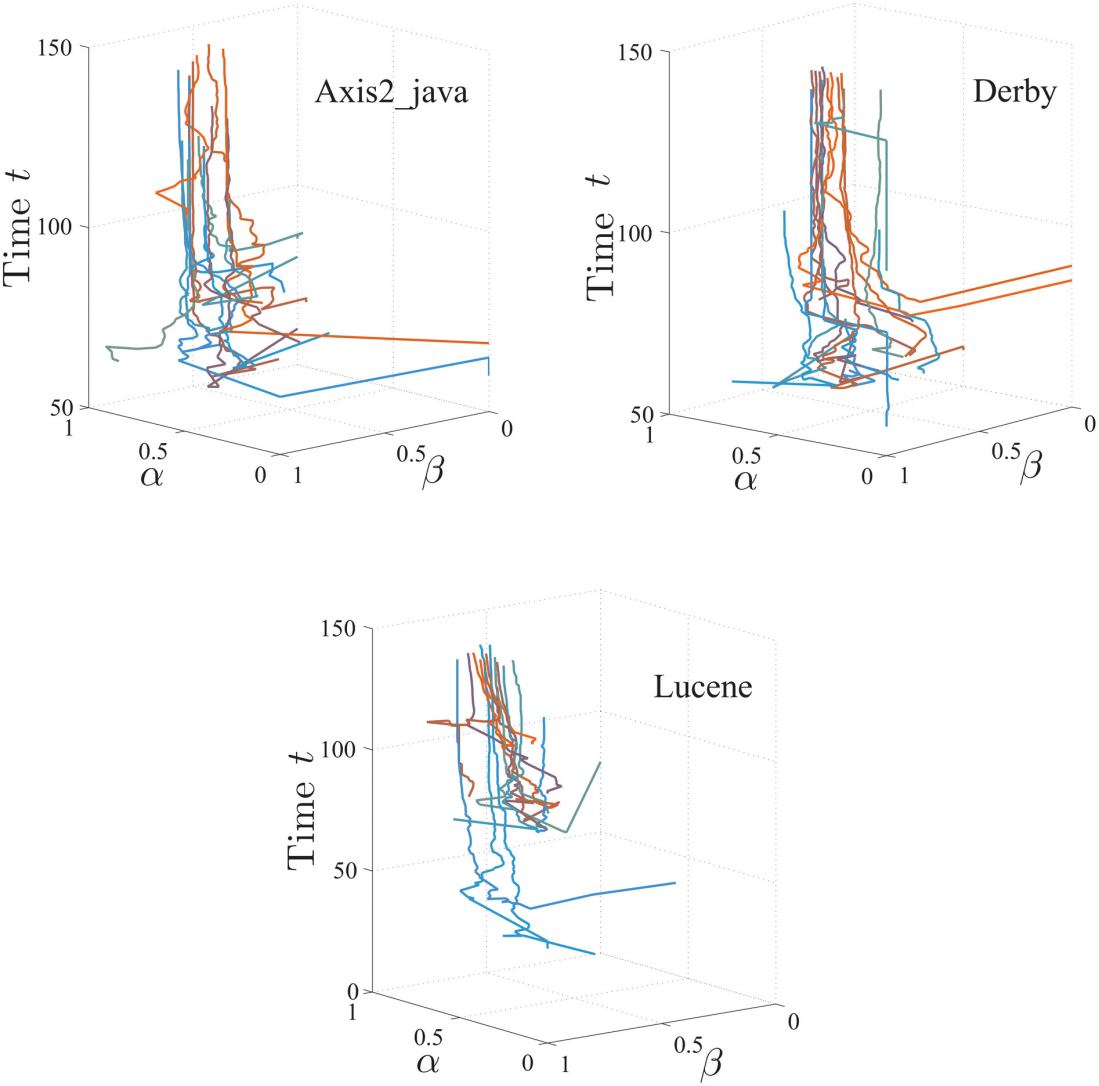
Developers’ *α* & *β* monthly evolving curves, e.g., *Axis2_java*, *Derby*, and *Lucene*.

To study the clustering phenomenon of W-T patterns in more detail so as to answer whether developers choose to join communities with similar W-T patterns as theirs or the similarity emerges over time as developers participate and evolve with their communities, we do the same pattern analysis as above, using only the initial 100 activities in the W-T sequences. Based on the comparison, we find that:

1. The developers in the same community showed similar W-T patterns starting with their inception into the project. I.e., for their first 100 activities, the distances of HMM parameters between pairs of developers in the same communities are significantly shorter (*p* = 3.1e-13) than those from different communities.
2. In addition, the community cultures of different communities converge rather than diverge from each other, as time evolves. I.e., both the inner (within-community) and inter (between-community) distances decrease significantly (*p* = 0) with time, as shown in Fig 6. We also calculate the average inner distance for all communities by considering their respective first *ϱ* activities with different values of *ϱ*, as shown in Fig 7, to study the converging process. We find that the inner distances decrease as *ϱ* increases, for most communities. As examples, the evolutions of the HMM parameters with time for the communities *Axis2_java*, *Derby*, and *Lucene* are shown in Fig 8.
3. The clustering of the HMM parameters within communities grows tighter with time. I.e., the convergence rates of the parameter distances from the first 100 activities to all activities within communities (the average distance decreases from 0.3381 to 0.1832) is significantly larger (*p* = 1.7e-7) than those between communities (it decreases from 0.4216 to 0.2861).

These findings suggest that developers with similar W-T patterns are indeed more likely to join in the same communities, and continue to harmonize their patterns as they work and talk as a team. In fact, since there are many online communities on similar topics, people can first experience the culture of these communities and then decide to join or not [41–43]. For OSS, it is clear that most developers do communicate a fair bit on the developer mailing list before actually contributing work [34, 44]; indeed, this type of “socialization” is a necessary pre-requisite to having one’s work contributions accepted. Thus, it is to be expected that the developers are more likely to join in the communities with harmonized work and talk patterns, in order to reduce co-ordination efforts.

In addition, we find that different community cultures will slightly converge rather than diverge from each other over time; this suggests that there may be an over-arching trend of the W-T patterns for all the developers (in all communities). To investigate this further, we compare the two parameters *α* and *β* separately for all developers, considering *a)* the first 100 activities and *b)* all activities. We find that both of them increase as time evolves, i.e., the HMMs in case *a)* have significantly smaller *α* (*p* = 0.027) and *β* (*p* = 1.4e-5) than those in *b)*. In fact, the efficiency of overall work and talk activities may be measured by the sum *α* + *β*; larger values of this sum indicate less switching between activities and thus fewer interruptions. This arguably represents higher efficiency [45–47]. In other words, the HMM parameters (*α*_*i*_, *β*_*i*_) shown in Fig 4 can be fitted by the linear function:

$$\begin{array}{c} \alpha +\beta =\varepsilon , \end{array}$$ (18)

with a single parameter *ε* representing the average efficiency of all the developers. Using the least squares method, we get the average efficiency *ε* and the corresponding standard deviation *σ* from the regression line as

$$\begin{array}{c} \varepsilon =\frac{\Sigma_{i=1}^{N}\left(\alpha_{i}+\beta_{i}\right)}{N},\;\sigma =\sqrt{\frac{\Sigma_{i=1}^{N}{\left(\alpha_{i}+\beta_{i}-\varepsilon \right)}^{2}}{2N}}, \end{array}$$ (19)

respectively, for the *N* developers. We find that the average efficiency steadily increases, while the variance decreases, with time, which means that as time goes on developers tend to have longer bursts of pure work and pure talk, suggesting that their discussions are becoming more effective, and that the ensuing co-operative work proceeds relatively more uninterruptedly.

Looking at the change in the rate of talk activities for all developers, in terms of *α* and *β*, Eq (8), we find that the rate increases significantly (*p* = 0.0046) with time, indicating that most developers become more socialized in the process. This phenomenon is consistent with the fact that more discussions are always needed to further improve a mature product. Meanwhile, contributing to these online communities is social work, i.e., the contributions of developers are highly visible and will be checked by many other users [33], so it is not surprising that they need to reply to comments more frequently when contributing more.

### Community Culture and Individual Performance

We then study the correlations with community culture of five measures of individual performance *work rhythm* (# work activities per day), *thousands of lines of code added per unit time* (KLoC per day), *talk rhythm* (# talk activities per day), *newly established social links per week*, and *observed survival time* (# year), resp., *X*_1_ to *X*_5_. The first four properties are calculated in the same time period of the person’s W-T sequence. The *survival time*, *X*_5_, of a developer is defined as the period of time from her first activity to the last one, which may be longer than the period of their W-T sequence, considering that the W-T sequences under study were preprocessed by removing prefixes of pure work or talk activities. The survival time of a developer is only observed when the developer has left the respective community. Here, as a reasonable estimation, we consider that a developer has left the community if they have not been active for a relatively long time, i.e., longer than some threshold *T*.

**Fig 9:**
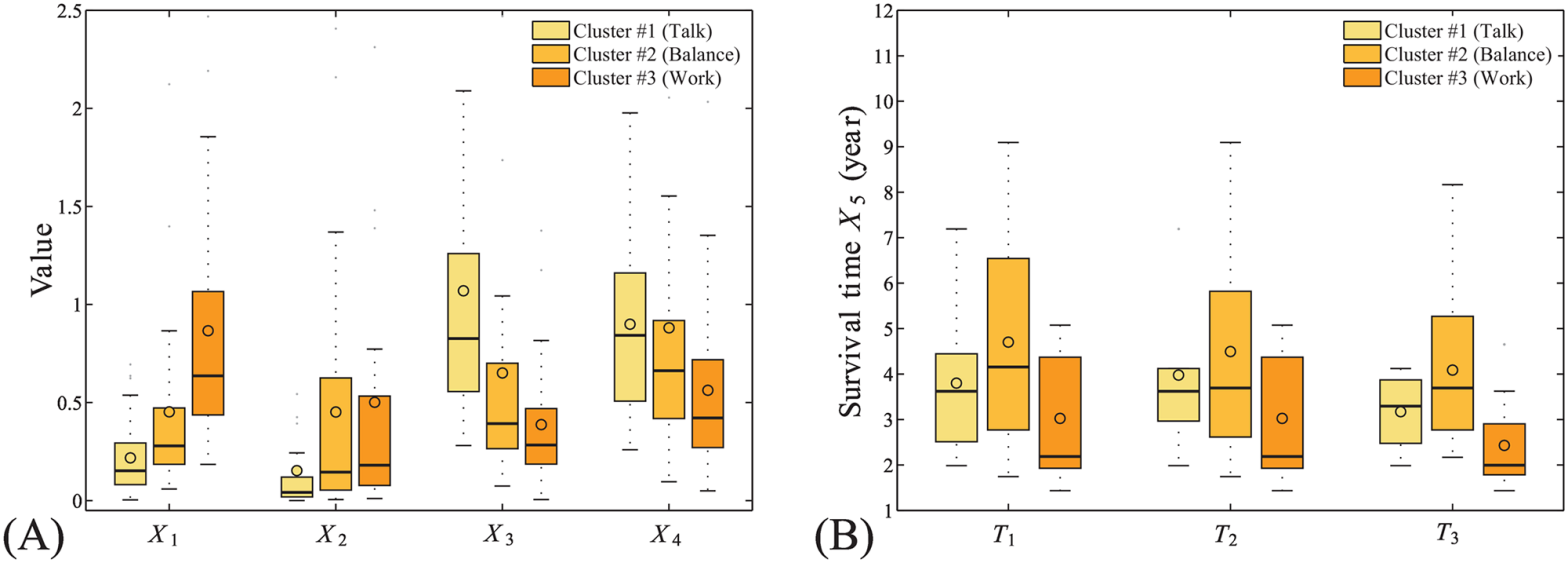
The effects of community culture on individual properties. The box-and-whisker diagrams for (A) the four individual properties *X*_1_ to *X*_4_, and (B) the observed survival time *X*_5_ with different time thresholds *T*_1_ (half year), *T*_2_ (one year), and *T*_3_ (two years), for the developers in the three clusters determined by their HMM parameters.

All developers are divided into three clusters by their HMM parameters, as shown in Fig 4. The developers in Cluster #1 emphasize “talk”, those in Cluster #3 emphasize “work”, while those in Cluster #2 seek balance between the two. For each property from *X*_1_ to *X*_4_, we have a list of their values for developers in each cluster, and the comparisons between the properties of developers in different clusters are visualized by the box-and-whisker diagrams shown in Fig 9(A), with the significance presented in Table 2. We find that the developers in Cluster #3 have the fastest working rhythms, those in Cluster #2 follow, while the developers in Cluster #1 work the slowest. The direction reverses for their talking rhythms. However, the situation is a little different when we compare the abilities of developers of different clusters in producing codes and earning social status. We find that the developers in Cluster #2 and Cluster #3 can produce similar KLoC per day, and both groups produce significantly more than the developers in Cluster #1, while the developers in Cluster #2 and Cluster #1 earn similar numbers of social links per week, and both groups earn significantly more than the developers in Cluster #3. Interestingly, balanced, and W-heavy W-T patterns are likelier among ASF members: the fractions of ASF members in Clusters #2 and #3 are 55.6% and 55.9%, respectively, while in Cluster #1 it is 46.3%. These indicate that extended discussion is always accompanied with the slowing down of work rhythms, but not always with decrease of productivity, and the developers seeking balance between work and talk behave competitively on both productivity and socialization as those who mostly work or mostly talk.

**Table 2:** The student’s t-tests for five individual properties between different clusters. Here, *p*_*ij*_ denotes the significance of difference between the developers in Cluster #*i* and Cluster #*j*.

Property	Mean value			Significance		
	#1	#2	#3	*p*_12_	*p*_23_	*p*_13_
*X*_1_	0.2173	0.4524	0.8664	0.0189	0.0054	1.23e-07
*X*_2_	0.2099	0.7955	0.7930	0.0142	0.9939	0.0146
*X*_3_	1.0696	0.6503	0.3878	0.0119	0.0576	6.63e-06
*X*_4_	0.8988	0.8814	0.5626	0.9035	0.0365	0.0020
*X*_5_	3.7974	4.6988	3.0243	0.3016	0.0368	0.2692

Although it seems that the developers who mostly work have the fastest working rhythms and the highest productivity, on average, it doesn’t mean that choice is the healthiest for them or for the overall community, since these developers are more likely to feel boring and then quit the communities. Based on the Hazard model to consider the censoring data, we find that developers with smaller *α* or larger *β* will have suggestively longer survival times (*p* = 0.077 and *b* = 1.7 for *α* and *p* = 0.042 and *b* = −2.4 for *β*), indicating that, by comparison, talk activities are more important than work activities for developer retention. Indeed, we find that developers with more balance between their work and talk stay active in the communities for suggestively longer periods of time than those who mostly work, as shown in Fig 9(B), i.e., the significance is equal to 0.037, 0.078, and 0.049 when the survival times of the developers with their last activities occurred half year, one year, and two years before are considered, respectively. The significance of comparison for the survival time among the three clusters of developers are presented in Table 2 when *T* = 0.5 (year). These findings suggest that developers with balanced W-T patterns are important to sustain OSS communities. Each of the communities we studied has at least one balanced developer, and there is also a natural trend that developers become more balanced, i.e., both *α* and *β* increase with time.

### Role of Socio-Technical Links

Here, we study the extent to which developers with similar W-T patterns tend to be linked more in the email social network or the technical cooperation network. In social networks, social weight between two developers intuitively means the number of emails between them. In cooperation networks, a pair of developers are linked with an edge indicating the number of files on which they have both worked. In particular, denoting by *ψ*_*i*_ the list of files that developer *d*_*i*_ commits to, the cooperative weight between a pair of developers *d*_*i*_ and *d*_*j*_, in terms of the files to which they have committed, is defined as

$$\begin{array}{c} \omega_{ij}=\frac{\psi_{i}\cap \psi_{j}}{\psi_{i}\cup \psi_{j}}. \end{array}$$ (20)

On the social side, for pairs of developers, we get *Spearman* correlations (*Pearson* correlations yield very similar results) between the distances of HMM parameters and the number of emails they have exchanged, shown in Table 3, in the Social weight columns. We find negative correlation in ten out of fourteen projects, with the significance *p* < 0.1 in six of them, including *Axis2_c*, *Camel*, *Derby*, *Lucene*, *Ode*, and *Solr*, while we find positive correlation with the significance *p* < 0.1 in only one project called *Mahout*. The negative correlation means that the smaller the HMM parameter distance between two developers, the larger the number of emails they have exchanged.

**Table 3:** Spearman correlation of HMM parameters and social & cooperative weights for developer pairs in different projects.

Project	Social weight		Cooperative weight	
	Correlation	Significance	Correlation	Significance
Activemq	–0.3056	0.2680	–0.5607	0.0323
Ant	0.0049	0.9774	–0.3704	0.0268
Axis2_c	–0.4667	0.0123	0.2474	0.2036
Axis2_java	–0.0442	0.6547	–0.1714	0.0805
Camel	–0.6000	0.0204	–0.3679	0.1779
Cxf	0.0651	0.7793	0.1948	0.3957
Derby	–0.1940	0.0337	–0.3232	3.41e-04
Lucene	–0.6046	2.20e-10	–0.2275	0.0303
Mahout	0.6685	0.0064	–0.3429	0.2110
Nutch	–0.2832	0.3065	0.4071	0.1333
Ode	–0.4866	0.0659	–0.1429	0.6114
Openejb	0.0667	0.8648	–0.3818	0.2790
Solr	–0.5083	0.0058	–0.5457	0.0031
Wicket	–0.1363	0.4876	–0.1795	0.3591
All	–0.2517	2.81e-09	–0.2037	1.84e-06

On the technical end, we study the *Spearman* correlation between the distances of HMM parameters and the strength of file cooperation links between developers. We get the results in Table 3, under the Cooperative weight columns. In this case negative correlation is found in eleven out of fourteen projects, with the significance *p* < 0.1 in six of them, including *Activemq*, *Ant*, *Axis2_java*, *Derby*, *Lucene*, and *Solr*, while no project has positive correlation with significance *p* < 0.1. The negative correlation means that the smaller the HMM parameter distance between two developers, the larger the cooperation between them.

When considering all communities together, we obtain a significantly negative correlation in both cases (the last row of Table 3). Thus, developers with more emails between them or committing to more of the same files are more likely to have similar W-T patterns. The results also indicate that community culture may be either social or task (technical) oriented; the distances between HMM parameters are more likely to be correlated with social weights in some communities, and with cooperative weights in others. Note that such findings are reasonable, considering that developers who commit more to popular files or who communicate more are likelier to coordinate more with each other [48], which may require higher-level convergence between their W-T patterns.

## Discussion

In this paper, we demonstrate that work-talk patterns of software developers in a number of OSS communities can be effectively studied using sequence analysis methods on sequences arising from simple two-state behavior models of work and talk activities.

Our methods enabled us to learn about a series of interesting task-oriented community based phenomena: that developers in a community present similar W-T patterns, and this clustering of W-T patterns is enhanced with time, reflecting different work cultures in these communities, with emphasis on different proportions of continuous work to continuous talk activities; that social and technical interactions may play a role in synchronizing W-T patterns, since developers with stronger social or technical links in a community have more similar W-T patterns; and that although successful task-communities may have relatively different cultures, developers with balanced work-talk patterns seems to play critical roles in sustaining them, and, at least in the ones we studied, each has at least one such developer. These findings suggest that online individuals may synchronize their behaviors with others to better fit in the task communities and to improve coordinating efficiencies. We acknowledge that the talk activities we discussed here are meaningfully related to work activities, and thus can be considered also as being work activities, in a more general sense. But commits and email communications are still different kinds of activities [28, 33, 49] and thus it’s reasonable to use HMM to describe the switching patterns between them. Moreover, it is because talk and work are related that the functional W-T patterns are meaningful.

In the future, the methods proposed in this paper can be further expanded and applied to analyze the switching pattern of more varied kinds of activities in more diverse online communities, such as GitHub [50], Wikipedia [51], and StackExchange [52]. Like other empirical research, our work is based on a sample of work and talk activities of developers, although commits [49, 53–55] and email communications [32, 56] have been extensively studied in the area of software engineering and were referred as *work* and *talk* [28], respectively. In reality, developers may have other kinds of work activities, e.g. consulting StackOverflow, which are relatively difficult to be captured. There are also talk activities that we do not capture, e.g., the discussion on issue tracking systems. Collecting more complete data sets will definitely help to derive more comprehensive results. To address this, we collected issue tracking data from Jira and Bugzilla [35], and arguably experimented with including them as talk activities (both opening issue as initializing the discussion and comments). Our result did not change significantly, indicating the revealed phenomena are quite robust. We use lines of code, LoC, to measure the productivity of a developer since it has been used extensively [27, 49, 53]. This, while appropriate, is not the only such measure. Alternative metrics include the number of issues fixed [57], the development time of tasks [58], and the number of bugs [59]. Using them may offer additional insight into the benefits of balanced W-T patterns.
